# Terahertz Humidity Sensing Based on Surface-Modified Polymer Mesh Membranes with Photografting PEGMA Brush

**DOI:** 10.3390/polym15153302

**Published:** 2023-08-04

**Authors:** Borwen You, Chih-Feng Huang, Ja-Yu Lu

**Affiliations:** 1 Department of Physics, National Changhua University of Education, No. 1 Jinde Road, Changhua 500207, Taiwan; borwenyou@cc.ncue.edu.tw; 2 Department of Chemical Engineering, i-Center for Advanced Science and Technology (iCAST), National Chung Hsing University, 145 Xingda Road, South District, Taichung 40227, Taiwan; 3 Department of Photonics, National Cheng Kung University, No. 1 University Road, Tainan 70101, Taiwan

**Keywords:** surface modification, photografting, degree of polymerization, polymer brush, terahertz, relative-humidity sensing, non-destructive detection

## Abstract

A simple and compact intensity-interrogated terahertz (THz) relative humidity (RH) sensing platform is successfully demonstrated in experiments on the basis of combining a porous polymer sensing membrane and a continuous THz electronic system. The RH-sensing membrane is fabricated by surface modification of a porous polymer substrate with hydrophilic and photosensitive copolymer brushes via a UV-induced graft-polymerization process. The intensity interrogation sensing scheme indicated that the power reduction of the 0.4 THz wave is dependent on the grafting density of the copolymer brushes and proportional to the RH percent levels in the humidity-controlled air-sealed chamber. This finding was verified by the water contact angle measurement. Based on the slope of the proportional relation, the best sensitivity of the hydrophilic surface-modified sensing membrane was demonstrated at 0.0423 mV/% RH at the copolymer brush density of 1.57 mg/mm^3^ grafted on the single side of the sensing membrane. The sensitivity corresponds to a detection limit of approximately 1% RH. The THz RH sensing membrane was proven to exhibit the advantages of low loss, low cost, flexibility, high sensitivity, high RH resolution, and a wide RH working range of 25–99%. Thus, it is a good candidate for novel applications of wearable electronics, water- or moisture-related industrial and bio-sensing.

## 1. Introduction

Relative humidity (RH) sensing becomes significantly important due to its broad applications in weather forecast, living environment control, quality control in the industrial production process, food processing, agricultural production, pharmaceutical processing, and civic engineering [1]. Demands for low-cost humidity sensors with high sensitivity, reliability, repeatability, and fast response time are large. Conventional RH-sensing methods include the electrical, optical, and mechanical sensing approaches, which are usually integrated with humidity-sensitive materials. A wide variety of hygroscopic materials have been demonstrated for RH sensing including ceramics [2,3], semiconductors [1,3], polymers [1,3,4,5], and biocompatible medium [6,7]. Particularly, various hygroscopic polymers, such as poly(vinyl alcohol) (PVA) [1,3,4,5] and polyimides [1,3], are developed for RH sensing with the advantages of low material and fabrication costs, ease of manufacture, high availability, and high flexibility. They are usually manufactured as porous forms or combined with porous structures to increase the active surface areas, to substantially increase the adsorbed moisture concentration and enhance RH sensitivity [3]. In general, these hygroscopic materials have RH-dependent variations in electrical, dielectric, or mechanical properties after chemisorption or physisorption of water vapors, caused by hydrophilic or hydrophobic property changes in the sensing surface. For example, the resistive-type humidity sensor responds with respect to conductivity change, whereas the capacitive-type and optical humidity sensors respond with respect to the permittivity change [3]. The mechanical-type humidity sensor responds to mass or volume change based on the swelling of the hygroscopic material, modulating the propagation of the surface acoustic wave [8]. Compared with electric and mechanical humidity sensors, the optical RH-sensing method has advantages of high resistances in electromagnetic interference and harsh environment, remote sensing capability, and not requiring frequent maintenance. However, optical RH-sensing devices, such as the fiber-type [1] and attenuated total reflectance-based humidity sensors [5], still struggle with optical scattering, long-term stability of the optical source, requirement of operating in a dust-free and stable environment, and complex sensing configuration.

The optical RH sensors operating at long-wavelength regimes, such as THz waves or microwaves, are implemented to solve these problems. For example, in a microwave regime, a metamaterial-type double-sided resonator structure with chickpea powders as the humidity-sensitive layer was demonstrated for frequency-interrogated RH sensing [6]. The best frequency shift sensitivity was experimentally demonstrated as 51.43 MHz/% RH at the X-band. However, it only works at low humidity levels within a small linear response range from 10.5% to 21.0% RH [6]. Another microwave RH sensor works on the basis of incorporating a sensing layer TOCN/PPy, i.e., conductive polymer-doped cellulose nanofibers, into a coplanar waveguide structure, demonstrating a proportional phase response to different RH levels from 22.8% to 75.3% [9]. The sensitivity of the phase-sensitive RH sensor, approximately 0.154°/% RH, was measured at 5.870 GHz but with a relatively high insertion loss of 1.26 dB [9].

In the THz regime, the field–analyte interaction strength, associated with the electric dipole moments [10] of molecules, is comparable to that of infrared, but is 10^3^–10^6^-fold stronger than microwaves. THz waves have particularly high sensitivities to many polar molecules, such as water and various polar gases, endowing THz RH detection [4,7,11,12,13] and sensing capabilities of various volatile organic compounds (VOCs) [10,12,14]. Thus far, publications on RH sensing in THz regimes are few. The THz RH-sensing platforms are classified into two major groups. One approach is based on intensity interrogation of water vapor fingerprints using the spectroscopic systems, including pulsed THz time domain spectroscopy (THz-TDS) [11,12,13] and continuous wave (CW) frequency domain spectroscopy (FDS), which was demonstrated by a photo-mixing technique [11]. THz waves have rich and crowded absorption characteristics of water vapors based on roto-vibrational energy level transitions [10,11,12,13,14] and a relatively high dipole moment of the water molecule, ~1.85 D [15]. However, the quantitative detection of humidity, utilizing the THz-wave probe, still relies on the long propagation length up to several tens of centimeters or meters in the ambient environment for enhancing the field–vapor interaction [11,12,13]. This sensing scheme is complex, ponderous, and disadvantageous to practical applications. For example, the moisture levels have been successfully identified in a high-pressure pipeline with a length of 14.7 cm based on the THz-TDS technique, and the limit of detection (LOD) was as low as 0.13% RH, equivalent to 62 ppm [13]. However, the linear response of RH is saturated beyond 23% [13]; that is, the spectroscopic sensing platform is only suitable for detecting at low humidity levels. The other THz RH-sensing approach is based on a frequency-interrogated resonant device integrated with hygroscopic materials, including the photonic crystal waveguide [4] (PCW) and metamaterial-based [7] THz photonic devices. The frequency-interrogated devices were designed to have one or several sharp spectral features in transmitted or reflected spectra, and their spectral shifts of resonances were susceptible to the refractive index changes of the hygroscopic layer caused by the RH variations of surroundings. For instance, the PCW-based THz humidity sensor with a hydrophilic PVA polymeric membrane as the sensing layer has been demonstrated based on the RH-dependent spectral shift and intensity variation at resonance frequencies [4]. The best sensitivity is exhibited at 70% RH with a frequency shift and a THz transmittance variation of approximately 0.25 GHz and 15% [4], respectively. A hybrid THz humidity sensor, consisting of a metamaterial and a silk protein fibroin film for moisture adsorption, has been demonstrated with a linear spectral shift at the resonant dip within 12.5–78% RH [7]. The sensitivity of the metamaterial-based THz humidity sensor was as low as 0.22 GHz/% RH with a LOD of 2.3% RH [7]. The THz resonant-type photonic devices, i.e., PCWs and metamaterials, have high-quality factors at resonantly spectral features and high sensitivities in membrane thickness detection [4,7]. However, they have poor RH sensing performance in spectral-shift sensitivity and LOD [4,7]. Therefore, the corresponding refractive index change, produced by adsorbing different amounts of water vapor on hygroscopic layers, is very small and insensitive to the THz resonant-type photonic devices.

In this study, an intensity-interrogated THz humidity sensor, composed of a poly(ethylene terephthalate) (PET) woven mesh substrate and a hydrophilic surface modification layer with macro-polymeric brushes, is proposed for RH sensing. The PET mesh-membrane substrate is surface-modified by the copolymeric chains or brushes with rich hydroxyl groups via UV-induced grafting and polymerization for effective water vapor adsorption. Nondestructively inspecting the dynamic formation of the hydrophilic polymer brushes, immobilized on a PET mesh-membrane substrate during the UV-curing process, is successfully demonstrated via in situ monitoring of THz transmittance by the THz-TDS technique. A simple CW THz electronic system based on a Gunn diode module [16] was used to characterize the sensing performance of the surface-modified membrane with different grafting densities of hydrophilic copolymer brushes. The qualitative and quantitative RH-sensing capabilities and the response time of the THz RH sensing membrane were investigated via interrogation of dynamic THz transmission, while exposing to different RH percent levels in a self-made air-sealed chamber. The detectable RH range with linear THz responses has been observed within 25–99% based on the available RH levels in an experiment. The best LOD of the THz RH sensor is as small as 1% for the sensing membrane, grafted with 1.57 mg/mm^3^ copolymeric brushes. For the portable RH-sensing platform, the current sensing modality based on the Gunn diode module can be replaced with a more compact THz quantum cascade laser. The simple, low cost, and flexible polymeric RH-sensing membrane has potential for future applications of electric skins, wearable electronics, and human–machine interfaces.

## 2. Materials and Methods

### 2.1. Synthesis of the Photosensitive and Hydrophilic Copolymer Brushes

**Figure 1:**
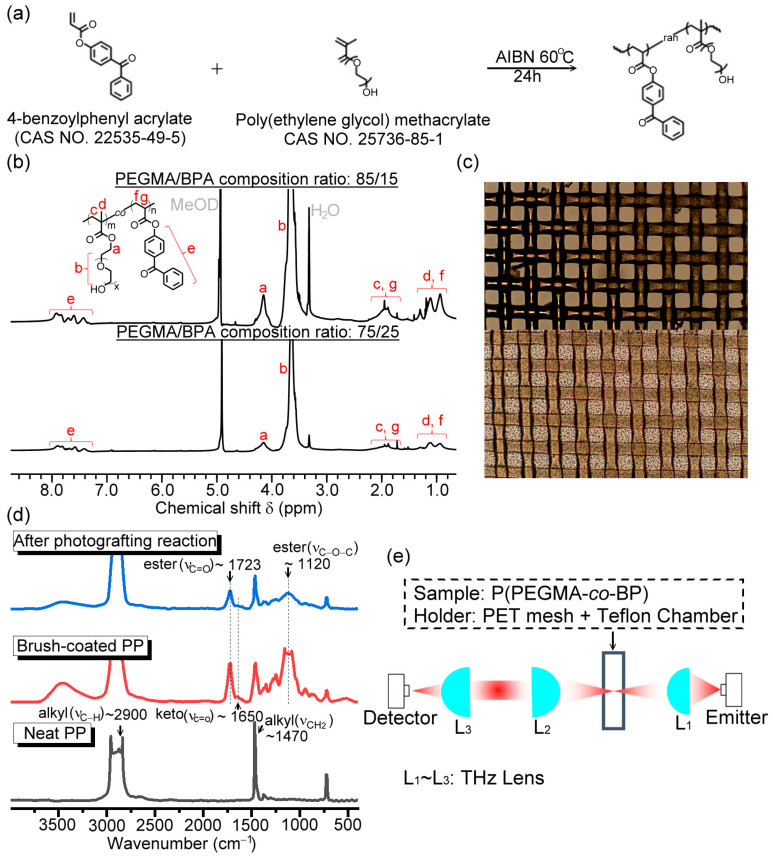
(**a**) Schematic process for the synthesis of P(PEGMA-*co*-BPA) macro-molecule based on the PEGMA and photosensitive BPA monomers. (**b**) ^1^H NMR spectra of the P(PEGMA-*co*-BP) copolymers. The PEGMA/BPA composition ratios of the copolymer are 85/15 (top) and 75/25 (bottom). (**c**) Microscopic photographs for a PET mesh membrane without (top) and with (bottom) hydrophilic surface modification. (**d**) FTIR spectra of a neat PP mesh (bottom), a copolymer brush-coated PP mesh before (middle) and after (top) photografting. (**e**) Schematically optical configuration of time-dependent THz measurement for in situ characterizing of the dynamic process of photografting and polymerization of the copolymer brushes on the PET mesh membrane under 365 nm UV irradiation in ambient conditions.

In this study, the specified macromolecule of Poly(PEGMA-copolymer-BPA), denoted as P(PEGMA-*co*-BPA), was copolymerized with the monomers of poly(ethylene glycol) methacrylate (PEGMA) (Merck Ltd., Taipei, Taiwan) and 4-benzoylphenyl acrylate (BPA) (Merck Ltd., Taipei, Taiwan). The BPA monomer functionalizes as the photo-initiator or photosensitizer for the copolymeric macromolecule, namely, P(PEGMA-*co*-BPA), whereas the PEGMA monomer shown in Figure 1a has the special structure of molecular brush [17,18] with a hydrophilic hydroxyl group for water vapor adsorption. The photosensitive and hydrophilic PEGMA-based copolymeric brushes were consequently generated by the repeated unit of PEGMA-grafted BPA polymer via a random cross-linking process in a solvent (Figure 1a). Particularly, the polymeric brushes based on P(PEGMA-*co*-BPA) could be immobilized and cross-linked to form a polymeric network layer with hydrophilicity on the substrate via a UV-induced graft-polymerization process.

In the synthetic process, BPA (0.75 g, 2.97 mmol), PEGMA (3.85 mL, 10.68 mmol), the initiator of azobisisobutyronitrile (AIBN, 0.05 g, 0.14 mmol) (Merck Ltd., Taipei, Taiwan), and their tetrahydrofuran solvent (THF, 24 mL) (Merck Ltd., Taipei, Taiwan) were mixed in a round-bottom flask. The flask was sealed and kept in an oil bath at the temperature of 60 °C for 24 h. The mixture was then concentrated and precipitated in methanol. Two monomer amount ratios of PEGMA/BPA were prepared, including 75/25 and 85/15, for the synthesis process and parameters to obtain P(PEGMA-*co*-BPA). For inspecting the copolymer molecules in proton nuclear magnetic resonance (^1^H NMR), the powders were collected, and dried in a 40 °C vacuum oven to remove the residual solvent. ^1^H NMR measurements were conducted in a deuterated solvent by using a Bruker Avance 400 MHz NMR spectrometer (Bruker BioSpin, Rheinstetten, Germany). Figure 1b shows the ^1^H NMR spectra of the obtained P(PEGMA-*co*-BP) copolymers with two composition ratios of 85/15 (top curve) and 75/25 (bottom curve). The chemical shifts δ of the copolymers were identified by using the Methanol-d_4_ (MeOD) (Merck Ltd., Taipei, Taiwan) as an internal standard (i.e., δ_MeOD_ = 4.78 ppm as shown in Figure 1b). The representative signals of the PEGMA unit (i.e., the peaks a–d) were assigned according to the pendant group and backbone (the inset of Figure 1b). The representative signals of the BPA unit (i.e., the peaks e–g) were also assigned. Although the signals of backbones are nearly overlapping (i.e., the peaks c and g, d and f), the copolymer compositions of PEGMA/BPA (i.e., the 75/25 and 85/15 values) can be clearly estimated from the spectral intensity ratio (i.e., I_peak a_/I_peak e_), indicating their simple and distinguishable chemical structures.

### 2.2. Fabrication of the Hydrophilic Copolymer Brushes on the PET Mesh Membrane

The substrate of the THz RH sensor with a low absorption loss of a broad THz spectrum is critical for efficiently sensing humidity with THz waves in experiments. Thus, a polymer woven-mesh membrane with a high open ratio of 30% and made of PET was used as the porous substrate of the THz RH sensor to integrate with the hydrophilic polymer brushes, whose mesh-opening size in a square unit and membrane thickness are 45 and 56 μm, respectively. The PET mesh is the product 120-34 Y PW, available from SEFAR PET 1500, Sefar AG, Heiden, Switzerland. The purchased PET mesh membrane is hydrophobic [18,19], which is verified by the water contact angle measurement. For RH-sensing purposes, the porous PET membrane was surface-modified by a hydrophilic layer via photografting the copolymer brushes on the membrane substrate. A P(PEGMA-*co*-BPA)-containing THF solution was dropped and uniformly dip-coated by a pipette on a circular PET mesh membrane substrate with a diameter of 22 mm. The photografting and polymerization processes were triggered by a 365 nm wavelength LED array (Model KO-M365-10, KOODYZ Technology Co., Xinbei City, Taiwan) with the illumination power of 10 W to immobilize and crosslink the copolymeric brushes to form a hydrophilic surface modification layer on the substrate. Figure 1c shows the photographs of surface-modified PET mesh membrane with and without grafting of PEGMA copolymer brush after 30 min UV exposure. The corresponding surface and hole space of the PET mesh are bulkily covered by the overlayer of P(PEGMA-*co*-BPA) copolymer brush (i.e., the bottom photo of Figure 1c). This finding indicates that P(PEGMA-*co*-BPA) copolymer brushes are completely polymerized and grafted to the PET mesh membrane, to successfully change the original hydrophobicity into hydrophilicity by dip coating a hydrophilic surface modification layer, which was verified by the water contact angle measurement in the following text. 

In the experiments, two composition ratios of PEGMA/BPA monomer in the P(PEGMA-*co*-BPA) copolymer brush, including 75/25 and 85/15, were synthesized and exposed to different durations of 365 nm UV light to study the effects of monomer concentration and grafting time on the extent of the photografting process via the THz-TDS technique [11,12,13]. THz-TDS in this study was used to monitor, in situ, the dynamic process of UV-induced grafting and polymerization of the hydrophilic copolymer brushes on the porous PET substrate. The BPA was initiated by the UV light illumination, and the P(PEGMA-*co*-BPA) copolymer brushes were subsequently anchored and grafted on the PET mesh membrane, possibly via the chemical affinity and binding of the functional group on the copolymeric brush, such as the methylene group, with various polar complementary groups on the PET substrate [3,18,19]. Once the P(PEGMA-*co*-BPA) copolymers were anchored to the substrate surface, the self-crosslinking or polymerization of copolymers would further occur at the grafting points to form the hydrophilic polymer network overlayer, which covers the holes and surface of the PET mesh membrane (i.e., the bottom photo of Figure 1c).

In order to verify the success of photografting and cross-linking the copolymer brushes on a polymer substrate, the P(PEGMA-*co*-BPA) copolymer layer was dip-coated on a polypropylene (PP) mesh to conduct the Fourier-transform infrared spectroscopy (FTIR) measurement before and after photografting. FTIR spectra were recorded by a Nicolet Avatar 320 FTIR Spectrometer with 32 scans at a resolution of 4 cm^−1^. To avoid the spectral fingerprints of a polymer substrate overlapping those of the copolymer brush, a PP mesh was chosen as the substrate instead of the PET woven mesh in the FTIR measurement. The PET material has a strong absorption peak at 1730 cm^−1^ from C-C ring stretching and C=O stretching [20], and can, thus, hinder the distinction of the spectral characteristics of P(PEGMA-*co*-BPA) copolymer brush near to 1730 cm^−1^. Figure 1d shows the FTIR spectra for the treatments of the P(PEGMA-*co*-BPA) copolymer brush on the PP mesh. In the case of the neat PP mesh (the bottom of Figure 1d), strong signals approximately at 2900 and 1470 cm^−1^ were ascribed to the PP mesh material, mainly comprising alkyl linkages (i.e., *ν*_C-H_: sp^3^ C-H stretching and *ν*_CH_2__: CH_2_ bending mode, respectively). In the case of brush-coated PP mesh (the middle of Figure 1d), the specimen significantly displays main characteristic peaks of ester (C(O)OC-) and keto (PH_2_C=O) groups of P(PEGMA-*co*-BPA) copolymer (i.e., 1723 cm^−1^ from PEGMA unit and 1650 cm^−1^ from BPA, respectively) [21]. In the case of after the photografting reaction (the top of Figure 1d), the peak of the ester group remained and the weak intensity of the keto group was further decreased [22]. After rinsing the irradiated mesh with THF in a purification procedure, the results indicate the formation of a crosslinked P(PEGMA-*co*-BPA) layer on the mesh frame and, based on a successful photografting reaction, through the BPA units.

### 2.3. Experimental Setups of THz Wave Systems

For preparing the hydrophilic surface modification of a sensing membrane and its RH-sensing performance, the related setups in experiments are presented in this section. Figure 1e schematically illustrates the THz optic configuration of a THz-TDS system for in situ THz waves, monitoring the dynamic photografting and polymerization of the PEGMA copolymer brushes on the PET mesh membrane under 365 nm UV irradiation in ambient conditions. The THz emitter and detector are one pair of photoconductive antennas (PCAs). The PCA is made of dipole electrodes on a low-temperature-grown GaAs substrate to perform 0.1–3 THz radiation emission and detection based on a femtosecond pulse laser excitation with a center wavelength of 780 nm. 

**Figure 2:**
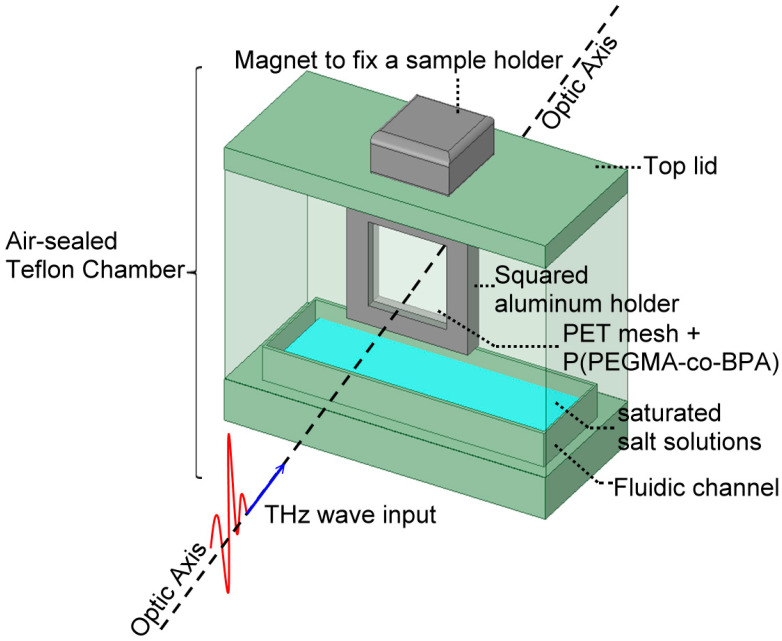
Configuration of THz RH measurement based on the PET mesh membrane with PEGAM polymeric brushes. The sensing device is packaged in a squared aluminum holder, fixed at the top lid of a Teflon chamber by a magnet and enclosed in the air-sealed chamber with a fluidic channel in the bottom infilled with a saturated salt solution.

For a humidity-sensing application, a THz RH-sensing device, composed of the hydrophilic surface-modified PET mesh membrane, was fabricated from the 85/15 composition ratio of the PEGMA/BPA formulation and enclosed in one self-made air-sealed Teflon chamber, to interact with THz waves. Figure 2 shows the configuration of THz RH measurement based on the surface-modified sensing device and inside a Teflon chamber. The sensing device is packaged in a squared aluminum holder, fixed at the top lid of a Teflon chamber by a magnet, and enclosed in the humidity-controlled chamber. Figure 2 shows one fluidic channel in this Teflon chamber to sequentially be injected by different saturated salt solutions and produce different RH levels inside the air-sealed chamber. Table 1 lists various RH percent levels at 25 °C and 1 atm, produced by the saturated salt solutions of CH_3_COOK, MgCl_2_, K_2_CO_3_, NaCl, and KCl [23]. In addition, the pure water would introduce 99% RH in the air-sealed chamber, whereas the ambient RH values of the laboratory, measured by a commercial electric hygrometer (COOLPLAY CP-501, Jiamen Ltd., Taoyuan City, Taiwan) in an opened Teflon chamber and at different dates, slightly fluctuate from 46% to 52% depended on the environmental conditions. The RH values of all of the saturated salt solutions and pure water in the chamber space were stable and repeatable while using the same electric hygrometer and chamber kits to obtain their RHs, because the chamber-sealing quality is sufficiently high. 

In the humidity-sensing experiment, a CW electronic THz system was used to characterize the sensing performance of the surface-modified PET mesh membrane. A 0.4 THz continuous wave was obtained from a Gunn-diode-based emitter module and detected by a Schottky diode [16]. Combining a lock-in amplifier and a mechanical chopper with the Schottky diode can enhance the signal-to-noise ratio of the detected THz wave power. The spatial arrangement of the CW THz electronic system for the RH-sensing experiment is similar to that of the pulsed THz spectroscopic system illustrated in Figure 1e, including the combination of one THz emitter, one THz detector, three THz lenses, and one target sample.

The THz RH-sensing experiment was conducted by intensity interrogation of 0.4 THz transmitted intensity from the surface-modified PET mesh membrane enclosed in the humidity-controlled air-sealed chamber. The power recording duration was sustained for 3500 s to observe the time-dependent THz response to investigate the dynamic performance of the THz wave power response to each RH condition. When the injected saturated salt solution is replaced in the air-sealed chamber for a specific RH level, the THz incident power at *t* = 0 s should be calibrated and maintained.

## 3. Results and Discussion

### 3.1. THz Characterization of the Copolymer Brushes on the PET Mesh Membrane

**Figure 3:**
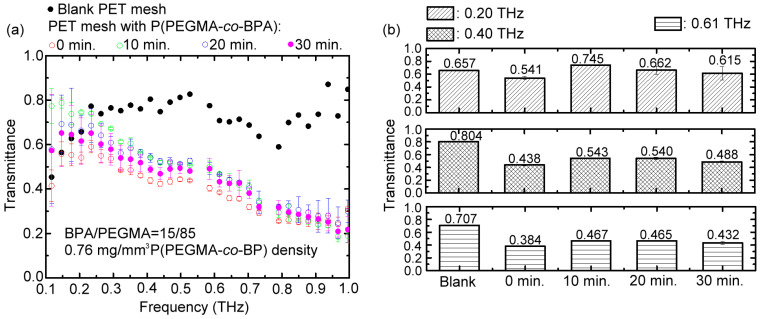
Transmittance of the surface-modified and blank PET mesh membrane (**a**) within 0.1–1 THz and (**b**) at the selected frequencies, which were dynamically measured at different UV-curing durations.

Figure 3a illustrates the measured THz wave transmittance of PET mesh membranes in 0.1–1 THz with and without surface modification of photografting polymer brush, P(PEGMA-*co*-BPA), whose PEGMA/BPA composition ratio is 85/15 and grafting density is 0.76 mg/mm^3^. The maximum UV illumination duration for photografting the polymer brush is 30 min. The timings to monitor the brush formation via THz wave transmittance are 0, 10, 20, and 30 min. Prior to photografting, only the blank PET mesh membrane was measured for the spectral transmittance. Compared with the surface-modified membranes, the THz transmittance of the blank PET mesh membrane is the highest because of the least absorption from the substrate materials. The spectral trend of blank PET mesh transmittance is evidently flattened due to the low loss porous structure, performing an average transmittance of 0.72. On the contrary, the transmittance of the surface-modified PET mesh membrane has an approximately negative correlation with THz frequency due to the overlayer of the P(PEGMA-*co*-BPA) polymer brush. Thus, high-frequency THz waves have relatively low transmittance due to the intrinsic absorption of the crosslinked polymer network composed of P(PEGMA-*co*-BPA) polymer brushes.

The interaction of the THz wave and P(PEGMA-*co*-BPA) macromolecule is based on the THz electric-field ($E_{THz}$)-induced molecular dipole moment ($p_{M}$) and $E_{THz}$-driven molecular rotation through the torque between $p_{M}$ and $E_{THz}$ [10,24,25]. The time varying electric field from THz radiation can be significantly absorbed to drive the dipole oscillation and molecular rotation for the free macromolecules [10,24,25]. Nevertheless, less THz power is depleted for the immobilized P(PEGMA-*co*-BPA) macromolecule than the uncured copolymer macromolecule because the surface-anchored force of the former is resistive to the $E_{THz}$-driven molecular rotation. This finding implies that the extent of intermolecular crosslinking among P(PEGMA-*co*-BPA) macromolecules can be identified via intensity interrogation of THz-transmitted intensity from the surface-modified PET mesh-membrane during the polymerization process.

Above inference can be validated by the following experimental results as shown in Figure 3. At the monitor timing of 0 min, the un-immobilized copolymer macromolecules of P(PEGMA-*co*-BPA), without intermolecular crosslinking, strongly absorb THz waves, consequently performing the lowest transmittance compared with those under the longer durations of UV exposure (Figure 3a). This condition indicates that the immobilization of P(PEGMA-*co*-BPA) on the PET mesh membrane via the photografting process with a UV-illuminated duration within 10–30 min reduces the THz field energy absorption, which is caused by the THz-driving molecular dipole oscillations of P(PEGMA-*co*-BPA) [10,21,22]. That is, the anchoring force of the copolymer macromolecule resists the resonantly dipolar oscillation. The surface-modified PET mesh after UV-induced graft polymerization has a relatively higher THz wave transmittance than that prior to UV-induced grafting.

The spectral curves in Figure 3a show the 0.20, 0.40, and 0.61 THz wave transmittances for the blank PET mesh membrane, and the different monitor timings of UV-induced graft polymerization are summarized in Figure 3b. The finding clarifies that the THz wave transmittance of the surface-modified PET mesh membrane increases to the maximum values within 10 min. However, the THz wave transmittance slightly reduces at grafting time within 10–30 min because the temperature of the surface-modified PET mesh slightly rises when absorbing UV light from 10 to 30 min. The thermal motion of the copolymeric macromolecule slightly lose the surface anchoring and consequently enhances THz electric field absorption even when immobilized. The experiment result in Figure 3 expresses that the duration of UV exposure time should not be less than 10 min for a complete graft polymerization process of P(PEGMA-*co*-BPA) copolymer brush. In addition, to detect immobilization of P(PEGMA-*co*-BPA), the sensing method based on intensity interrogation of the THz transmitted signals is better than the proposed phase interrogation method [5,9] because the measured phase variation of the transmitted THz pulse from the ultra-thin immobilized layer during the photograft polymerization is extremely small and ignored.

**Figure 4:**
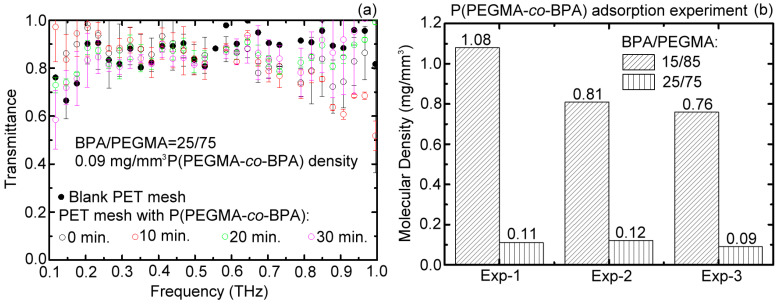
(**a**) Transmittance of surface-modified and blank PET mesh membrane within 0.1–1 THz, which was dynamically measured at different UV-curing durations. (**b**) Adsorbed copolymer densities observed in three experiments based on 75/25 and 85/15 monomer amount ratios of PEGMA/BPA.

The P(PPEGMA-*co*-BPA) overlayer is further used to surface-modify the PET mesh membrane with the 75/25 composition ratio of PEGMA/BPA and grafting density of 0.09 mg/mm^3^. The experimental result of the broadband THz transmittance spectrum is shown in Figure 4a measured by the same optical configuration (Figure 1e). Within the available THz range of 0.1–1 THz, the measured THz wave transmittances of the copolymeric brush-coated PET mesh membranes (Figure 4a) are indistinguishable among different UV exposure durations based on the system accuracy, but are considerably higher than those in Figure 3a. The result in Figure 4a reveals that the 0.09 mg/mm^3^ grafting density of the copolymer brush with the 75/25 composite ratio of PEGMA/BPA monomer on the porous membrane is too small to be detected by the THz probe and is independent of the UV exposure duration. Experimental tests (i.e., Exp-1, -2 and -3) were conducted three times for the 85/15 and 75/25 composition ratios of PEGMA/BPA samples under 15 min UV exposure duration to ensure the completeness of graft polymerization. The grafting density of the copolymer brush for each sample can be obtained by comparing the sample weight with and without the graft-polymerized P(PEGMA-*co*-BPA) brushes. The experimental results are summarized in Figure 4b, showing that the amount of copolymer brushes formed by the 75/25 composition ratio of PEGMA/BPA monomer cannot sufficiently be grafted to PET mesh membranes to absorb THz wave power. The best composition ratio of PEGMA/BPA was experimentally validated at 85/15 to form a surface-modified layer with a sufficient amount of copolymer brushes that were graft-polymerized on the PET mesh membrane. It eventually provides a vast number of hydrophilic hydroxyl groups for water vapor adsorption and endows one sensor with an excellent humidity-responsive property.

### 3.2. RH-Sensing Analysis

Based on the experimental result of frequency-dependent THz absorption for a porous surface-modified membrane as shown in Figure 3, the 0.4 THz wave was specifically applied to sense humidity in the Teflon chamber because of the relatively high transmittance decrement based on the response of the P(PEGMA-*co*-BPA) molecule on the PET mesh membrane, which is compared with those of the 0.20 and 0.61 THz waves in Figure 3. This finding indicates that the strong power absorption effect of P(PEGMA-*co*-BPA) molecule occurs at 0.4 THz frequency and that the 0.4 THz wave is sensitive to surface variation for the water adsorption with P(PEGMA-*co*-BPA) molecules.

**Figure 5:**
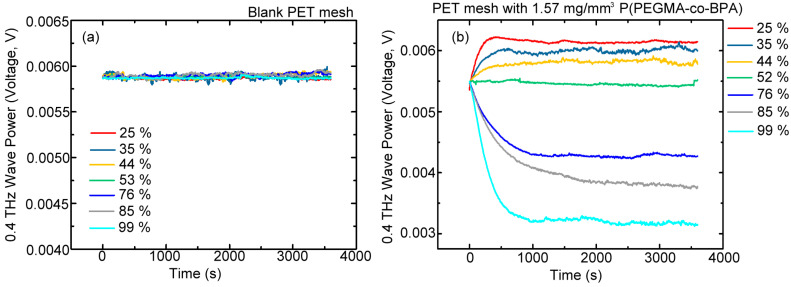
Measured transmission power of 0.4 THz wave from (**a**) a single layer of blank PET mesh membrane, and (**b**) PET mesh membrane grafted with 1.57 mg/mm^3^ copolymer brush under various RH percent-level exposures.

Figure 5a shows the measured time-dependent 0.4 THz transmitted power of one layer of the blank PET mesh membrane, exposing to different RH percent levels (25–99%, Table 1) inside the air-sealed chamber (Figure 2). However, no power change is found among all of the RH levels during one period of 3500 s. This result indicates that various RH levels cannot be distinguished from the transmission power curves in Figure 5a because of the approximate power voltage value. For example, for the 52% RH, the 0.4 THz wave sensing power is 0.0058 V on average, but other RHs are unidentified with detectable power difference. This finding reveals the consequence that the blank PET mesh membrane is hydrophobic and inactive to the environmental moisture [18,19]. Under the same THz wave-sensing scheme and RH conditions (Figure 1c and Figure 2, and Table 1), this blank PET mesh membrane was further replaced with the surface-modified one for dynamic transmission power measurement of 0.4 THz wave, and the result is illustrated in Figure 5b. In the experiment, the coated molecular density of the P(PEGMA-*co*-BPA) copolymer brush is 1.57 mg/mm^3^, contrary to the blank PET mesh membrane with 0 mg/mm^3^ brush density. For measuring 52% RH of ambient laboratory humidity, no aqueous solution injection found, and the chamber was sealed. The responding voltage level of the 0.4 THz wave power in Figure 5b is almost flattened with a constant voltage of approximately 0.0055 V on the average within the 3500 s recording duration. The 0.4 THz wave power from the 1.57 mg/mm^3^ surface-modified mesh membrane is decreased to approximately 5.8% compared with that of the blank PET mesh membrane (Figure 5a). The decreased THz transmittance resulted from the overlayer of P(PEGMA-*co*-BPA) copolymer brush that was grafted on the PET mesh membrane. It eventually changes the hydrophobic surface property as hydrophilicity and increases the adsorption amount of water vapor, thereby depleting the 0.4 THz wave transmitted power.

When the RH of the chamber is lower than 52%, the 0.4 THz transmitted power is gradually raised within 0–500 s and approaches to saturation with an individual constant power voltage higher than that of 52% RH, as shown by the responding curves of 25%, 35%, and 44% RHs in Figure 5b. The amounts of adsorbed water vapor at low humidity (i.e., 25%, 35%, and 44% RHs) are smaller than those at 52% RH, decreasing the absorption loss. In this condition, the water vapor was captured by the hydroxyl groups of the polymer chains or brushes grafted on the membrane substrate. Therefore, the relatively low RH results in relatively high THz transmittance. On the contrary, the 0.4 THz transmitted power gradually decreased within the initial 1000 s for the high RHs from 76% to 99%, and then the power trend became saturated nearly after 1500 s. For the microscopic version, the saturation of the power response curve expresses that adsorption and desorption of water vapor on the overlayer of the polymer brush approach equilibrium, and approximately 1000–1500 s is required to gradually achieve a steady power response (Figure 5b). The high humidity accordingly leads to great THz power reduction because the large surface and volume densities of water vapor were physically adsorbed on the surface and infilled into holes of the hydrophilic mesh membrane (Figure 5b). The result in Figure 5b indicates that the measured power levels of different RHs (25–99%, Table 1) in their saturation durations of response curves are significantly correlated to the adsorbed amounts of water vapor, which are proportional to the RH values. Additionally, the dynamically time-dependent curve of the THz transmission intensity within 3500 s exactly represents the interactive responses among the THz wave, surface-modified PET mesh membrane, and its environmental humidity, showing capability to quantitatively identify RH levels inside the air-sealed Teflon chamber.

The power difference of the saturated 0.4 THz waves, ΔV, between the test RH and the ambient RH could be used as the quantitative indicator for the hydrophilic adsorption of water vapor on the sensing mesh membrane. The negative (positive) power difference (ΔV) that represents the quantity of water vapor adsorbed on the hydrophilic mesh membrane at a specific RH is greater (less) than that at the ambient RH. The humidity-sensing experiment conducted for different polymer brush densities was separately operated at different days, and the relevant ambient laboratory RH is slightly deviated from 52% with an RH range of 44–52%, depending on the ambient temperature and pressure. Therefore, the saturated transmission power from a sensing membrane grafted with one polymer brush density at ambient laboratory RH should be measured as the reference power signal before each sensing experiment conducted on different days.

The power difference of the saturated 0.4 THz waves, ΔV, between the test RH and the ambient RH could be used as the quantitative indicator for the hydrophilic adsorption of water vapor on the sensing mesh membrane. The negative (positive) power difference (ΔV) that represents the quantity of water vapor adsorbed on the hydrophilic mesh membrane at a specific RH is greater (less) than that at the ambient RH. The humidity-sensing experiment conducted for different polymer brush densities was separately operated at different days, and the relevant ambient laboratory RH is slightly deviated from 52% with an RH range of 44–52%, depending on the ambient temperature and pressure. Therefore, the saturated transmission power from a sensing membrane grafted with one polymer brush density at ambient laboratory RH should be measured as the reference power signal before each sensing experiment conducted in different days.

**Figure 6:**
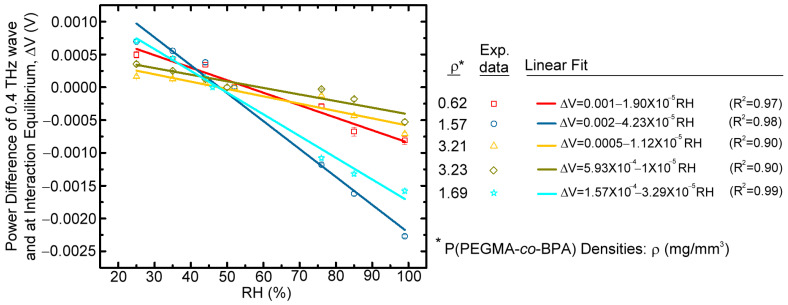
THz-transmitted power differences relative to the ambient RH condition for the sensing membranes coated with various copolymer brush densities. The measured results are shown as the data points, and their linear regression curves are represented as solid lines.

In this humidity-sensing experiment, two surface modification approaches use the P(PEGMA-*co*-BPA) copolymer brush: single-side surface modification and double-side surface modification. In the single-side surface modification, the solution of copolymer brushes was dip-coated on one side of the surface of a PET mesh membrane with a circular range of 22 mm diameter. Similarly, in the double-side surface modification, the solutions of the copolymer brushes were individually dip-coated on both (bottom and up) surfaces of one PET mesh membrane with the same circular area and location. The double-side surface modification was further treated once and twice with UV curing to immobilize copolymer brushes on the PET mesh membrane. The one-time UV curing with a 900 s duration is operated after dip-coating copolymer brush solution on the two sides of the PET mesh membrane, and the immobilized polymer brush density is 3.21 mg/mm^3^ (Figure 6). For longer curing duration, surfaces on both sides of the PET mesh membrane were individually dip-coated and cured, respectively, by copolymer brush solutions and UV illumination (900 s, 10 W), corresponding to the two-time UV-curing operation. The immobilized copolymer brush density of the two-time UV-curing operation is 3.23 mg/mm^3^ (Figure 6), which is slightly higher than that of the one-time UV operation.

However, their sensitivities, as shown in Figure 6, are approximate and much lower than that of single-side surface modification. Although the polymer brush density of a single-side surface-modified PET mesh membrane is much lower than that of the double-side surface-modified one, the sensitivity of the single-side modification is much higher than that of the double-side modification. The results in Figure 6 indicate that the quantities of active sites for effective adsorption of water vapors on the single-side surface-modified membrane are greater than those on the double-side surface-modified one. The low amounts of water vapor active sites that mainly resulted from the grafted and cured polymer brushes on the first-side surface are re-dissolved into the additional dip-coated THF solution on the other (second)-side surface of the porous PET membrane substrate (Figure 1c). The hydrophilicity based on the process of double-side surface modification weakens to adsorb water vapor due to the decreased amount of hydrophilic polymer brushes. Moreover, the double-side surface-modified membrane decreases the hydrophilicity when the UV-exposing intensity of the UV-curing process on the unit surface area is insufficient for the sensing membrane with a brush density of 3.21 mg/mm^3^, consequently reducing the quantity of crosslinked polymeric brushes that possess hydroxyl groups.

The humidity-sensing performance of the sensing membranes with different brush densities (Figure 6) is summarized in Table 2, including the sensitivity (*S*) and linearity (*R^2^*) (i.e., slopes and the coefficient of determination of a linear fitting curve), ΔV inaccuracy (*δα*) in measurement (i.e., an error bar scale), detectable RH range (*R*), and measured resolution of RH (*C*) (i.e., the limit of detection, LOD). In addition, the sensitivity can also be represented by the power difference ratio (ΔV/V), defined as $(V_{25\%RH}-V_{99\%RH})/V_{99\%RH}$, where $V_{25\%RH}$ and $V_{99\%RH}$ are the THz transmission power through the RH sensor at 25 and 99 %RH, respectively. The results in Table 2 show that the sequence of humidity sensitivity for different brush densities grafted via single- and double-side surface modifications, denoted as single and double in the parentheses, is 1.57 (single) > 1.69 (single) > 0.62 (single) > 3.21 (double) ≅ 3.23 (double) mg/mm^3^. The best sensitivity occurs at the brush density of 1.57 mg/mm^3^ grafted on the one surface of a PET mesh membrane, and the LOD of the RH value is approximately 1%, which was estimated from the slope (*S*) and ΔV inaccuracy (*δα*) (Table 2) within the detectable range of 25–99%. This result indicates that an overly high or an overly low brush density can reduce the hydrophilicity of the sensing membrane.

For the single-layered deposition of polymer chains on the mesh membrane, increasing the polymer brush density can raise the quantities of hydroxyl groups to adsorb more water vapor. As a result, humidity sensitivity, such as the sensitivity sequence of 1.57 (single) > 0.62 (single) mg/mm^3^, increases. However, an overly high grafting brush density would increase the thickness of the hydrophilic surface modification layer and reduce the adsorption efficiency of water vapor. This condition is probably due to the hydrophilic active sites of the polymer chains that are buried in the deep and inner section of the surface-modified layer to hinder water vapor diffusion and binding [26]. For example, the depressed sensitivities were observed in the following conditions: the double-side surface modification of polymer brush and the single-side surface modification of a high brush density (e.g., 1.69 mg/mm^3^). The most effective hydrophilic binding always occurs at the outermost surface, but the adsorption efficiency of water vapor decreased with the increasing layer thickness [26].

**Figure 7:**
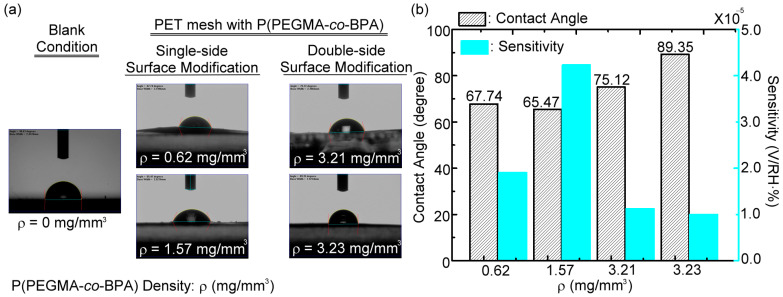
(**a**) Water contact angles of the PET mesh membrane without and with surface modification, including four densities of copolymer brushes. (**b**) Relations among the copolymer brush density, water contact angle, and RH detection sensitivity.

To validate the conjecture, the water contact angle measurement was further conducted for the mesh membrane without and with hydrophilic surface modification by four types of polymeric brush densities, as shown in Figure 7a. The sequence of contact angle for the mesh membranes grafted with different brush densities is pristine > 3.23 > 3.21 > 0.62 > 1.57 mg/mm^3^, which is opposite to the sequence of humidity sensitivity, as shown in Figure 7b. The pristine PET mesh membrane is hydrophobic with the largest water contact angle of ~90.63°, and the membrane with PEGMA brush density of 1.57 mg/mm^3^ has the smallest contact angle of ~65.47°. The smaller contact angle represents the more hydrophilicity of surface for water vapor adsorption and, thus, with a high humidity sensitivity.

**Figure 8:**
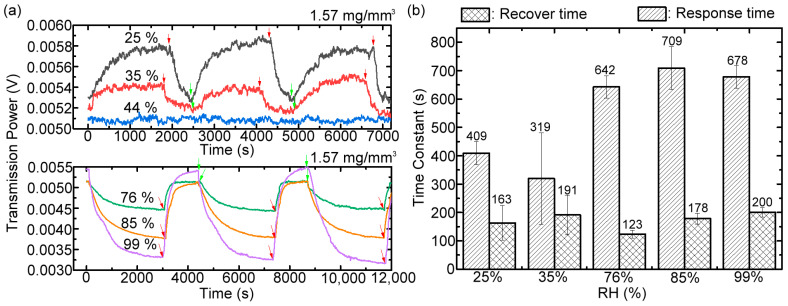
(**a**) Dynamic and repeatable THz power response curves for the RH-sensing membrane possessing copolymer brushes of 1.57 mg/mm^3^ measured by alternating cycling test RH and ambient RH of 46%. The test RH levels are 25%, 35%, 44%, 76%, 85%, and 99%. (**b**) Response and recovery times of the THz RH-sensing membrane under various RH-level exposures, which were fitted from (**a**).

Figure 8a shows the dynamic RH-sensing performance of the sensing membrane grafted with 1.57 mg/mm^3^ brush density for three continuous cycles of humidity exposure and exhaustion. This membrane for sensing humidity was enclosed into the humidity-controlled Teflon chamber with different saturated salt solutions (Figure 2, Table 1) and measured for its dynamic power transmission of the 0.4 THz wave, where the 46% RH is the ambient laboratory humidity. The dynamic recording of the THz-transmitted intensity from the sensing membrane starts when the saturated salt solution was injected in the air-sealed chamber at 0 s. During the sensing process, the water vapors are diffused, and the chamber is filled up and adsorbed on the sensing membrane until equilibrium, indicating the steadiness of the THz transmission power, as shown in Figure 8a. While the steady state of measured THz wave power is achieved, corresponding to the equilibrium of humidity exposure, the air pump is turned on to drain the adsorbed moisture of the mesh membrane, as indicated by the red arrows in Figure 8a. When the THz transmitted power achieved stability once again, corresponding to the equilibrium of humidity exhaustion, the air pump was turned off, as indicated by the green arrows in Figure 8a. Then, the second cycle of humidity exposure starts for water vapor adsorption on the sensing membrane. The three repeated measurements for each RH have almost identical THz transmission power at steady states. This finding reveals that the interaction between water vapors and the hydrophilic surface, grafted with polymeric brushes, belongs to physical adsorption, and the capability of repeatable usage performs without any degradation of the sensing performance.

When the RH level in the Teflon chamber was at 44%, approximating to the ambient humidity of 46%, there was no evident variation of the transmitted THz power from the surface-modified mesh membrane within the three periods of humidity exposure and exhaustion. The transmitted THz power increased/decreased at the low/high humidity exposure, i.e., below/above the 44% RH, whose power variation trend is similar to that of the humidity-sensing results in Figure 5b. The response and recovery times of the sensing membrane at different RHs, except for the ambient humidity, are illustrated in Figure 8b. The results were obtained by averaging the exponential fits of the THz transmitted power curves for three cycles of humidity exposure and exhaustion in Figure 8a. At the low RH (≤35%), the water vapors are fast bonded with the vacant hydroxyl groups of the polymer brushes grafted on the membrane surface; thus, the response time is short. On the contrary, for the high RH (>75%), more vaporized water molecules existed in the chamber space, and they not only fully occupied the hydroxyl groups on the outer surface of membrane, but they also further diffused into the deeper depth of the membrane for hydrophilic adsorption, increasing the response time with an inaccuracy of 50–100 s (Figure 8b). The recovery times of the sensing membrane (Figure 8b), ranging from 123 to 200 s, are independent of the relative humidity and only associated with the exhausted speed of the air pump (23 ℓ/min).

The slow response time can be further improved in two aspects, including the porous polymer substrate and the humidity-sensitive layer. For the porous polymer substrate, increasing the surface area or porosity of the surface-modified mesh membrane or replacing the hydrophobic substrate with a hydrophilic one, such as the polyamide (PA) porous membrane, can greatly increase the number of moisture-adsorption sites to shorten the response time. For the humidity-sensitive layer, decreasing the thickness of a brush-coated layer can lower the diffusion resistance of water vapor within this solid layer [7,26], which is validated in the experimental results of Figure 6, and, thus, the speed of water vapor adsorption rises. Additionally, hybridizing inorganic moisture adsorbents in the humidity-sensitive layer could also increase the adsorption efficiency of water vapor. 

Table 3 presents the sensing performance of the proposed sensor in this work, which is compared with the recently demonstrated polymer-based humidity sensors. This summary reveals that our proposed sensor is competitive with those RH sensors in sensitivity, detectable RH range, and RH resolution. A high linearity of 98% among RH-dependent THz signals has been verified within a wide RH range. Moreover, our proposed sensor also has advantages of simple configuration, compactness, flexibility, low cost, and an easy fabrication process.

## 4. Conclusions

In conclusion, a simple and flexible THz RH sensor, composed of a PET-woven mesh membrane with a hydrophilic surface modification layer, is successfully demonstrated to combine a compact CW THz electronic system for identifying different RH levels. The porous PET membrane is surface-modified by graft polymerization of the dip-coated P(PEGMA-*co*-BPA) copolymer brushes under UV exposure in ambient air. Two formulations of hydrophilic and photosensitive P(PEGMA-*co*-BPA) copolymer were synthesized in the THF solvent via chemically grafting different composition ratios between the brush-shaped PEGMA and photosensitive BPA monomers in which the PEGMA brush has a hydrophilic hydroxyl group for water vapor adsorption. The feasibility for nondestructively characterizing UV-induced grafting and polymerization of the hydrophilic polymer brushes with different monomer composition ratios on the PET mesh membrane has been demonstrated via the in situ THz-TDS technique. The saturation response of THz-transmitted intensity curves validated that the graft polymerization process is accomplished within the 15 min UV exposure duration, and the amount of grafted P(PEGMA-*co*-BPA) copolymer brushes increases by increasing the PEGMA–monomer concentration in the copolymeric formulation. The best monomer composition ratio of PEGMA/BPA was experimentally proven at 85/15 to graft polymerize a sufficient amount of copolymer brushes on the membrane substrate, consequently providing vast number of hydrophilic hydroxyl groups for water vapor adsorption and endowing the sensor with an excellent humidity-responsive property. The RH-sensing experiment, utilizing the copolymer brush-coated PET mesh membranes, was conducted in the self-made air-sealed Teflon chamber with different RH levels produced by the saturated salt solution method. The sensing result validates that the power reduction of the 0.4 THz wave is proportional to the RH percent levels within a wide RH range of 25–99%, which was fitted by a linear regression curve with high linearity (i.e., the coefficient of determination) of up to 0.98. The RH-dependent decrease in the THz-transmitted intensity resulted from the THz absorption loss of water vapors adsorbed on the copolymer brushes. In the experiments, the sensitivity of the RH-sensing membrane, represented by the slope of the linear regression curve, is correlated to the grafting density of the copolymer brushes on the sensing membrane. For the single-layered grafting of copolymer brushes on the sensing membrane, increasing the grafting density of copolymer brushes increases the hydrophilicity of the surface-modification layer as well as the quantities of water-adsorption sites. As a result, the relevant sensitivity increases, which is verified by water contact angle measurement. The best sensitivity using the intensity interrogation sensing scheme was 0.0423 mV/% RH at 0.4 THz for the brush density of 1.57 mg/mm^3^ grafted on the single side of PET mesh membrane, and its LOD in the RH value is approximately 1%. The dynamic sensing performance of the best sensing membrane under three continuous cycles of the humidification and dehumidification process was also characterized in situ by a CW THz system. The finding manifests that no hysteresis of THz-transmitted intensity occurs and shows the capability of repeatable usage without degradation of the sensing performance. The average response and recovery time of the RH sensing membrane were measured within durations of approximately 9 and 3 min, respectively. The slow response time can be further improved by increasing the mesh areas or hybridizing inorganic moisture adsorbents. The relevant studies are currently underway and will be demonstrated in near future. The simple and compact THz RH sensing platform based on the low cost and flexible surface-modified polymer membrane was experimentally demonstrated with high sensitivity and RH resolution in a wide RH range, showing great potential for applications, such as wearable electronics and water or moisture-related bio-sensing.

## Figures and Tables

**Table 1:** Different RH levels prepared from a series of saturated salt solutions [23].

Saturated Salt Solutions	Standard RH Values (%) *	Measured Standard RH Values (%) *
CH_3_COOK	22.5	25
MgCl_2_	32.8	35
K_2_CO_3_	43.2	44
NaCl	75.3	76
KCl	84.2	85
Water	—	99

* The temperature for those RH values is 25 °C.

**Table 2:** List of THz RH-sensing performance for the sensing membranes based on various copolymer brush densities.

Sensing Parameters	P(PEGMA-*co*-BPA) Density (mg/mm^3^)				
	0.62	1.57	1.69	3.21	3.23
S (V/RH%)	−1.90 × 10^−5^	−4.23 × 10^−5^	−3.29 × 10^−5^	−1.12 × 10^−5^	−1.00 × 10^−5^
S (ΔV/V)	30%	96.8%	52.8%	23.3%	23.7%
δα (V)	3.57 × 10^−5^	3.96 × 10^−5^	3.30 × 10^−5^	3.61 × 10^−5^	3.53 × 10^−5^
R (RH%)	25–99	25–99	25–99	25–99	25–99
C (RH%)	1.9	1.0	1.0	3.2	3.5
R^2^	0.97	0.98	0.99	0.90	0.90

**Table 3:** Summary of recently reported polymer-based humidity sensors.

Substrate	Sensing Layer	Sensing Mode	RH Range	RH Resolution	Sensitivity	Response/ Recover Time	Ref.
PET (Coplanar waveguide)	TOCN/PPy	Phase	22.8–75.3%	-	0.154°/% RH@5.87 GHz	-	[9]
PDMS (Guide mode resonant structure)	PVA	Transmission/ Frequency	6–70%	-	15% (ΔT)/0.25 GHz(Δf) @70% RH	-	[4]
Fused silica (Fiber based MZI)	PVA	Wavelength	35–95%	0.03%	0.6 nm/% RH	1.33 ms/2.01 ms	[27]
Silicon metamaterials	Silk protein fibroin	Frequency	12.5–78%	2.30%	0.22 GHz/% RH	Thickness- dependent	[7]
Borosilicate	Porous scandium polyacrylate	Transmission	10–90%	-	2.1 μW/% RH	25 s/155 s	[28]
Nanofibrillated cellulose (NFC)/CNT composite film	NFC/MWCNTs	Current	11–95%	-	69.9% (ΔI/I_0_)	330 s/377 s	[29]
PET	MWCNT/hydroxyethyl Cellulose	Resistance	20–80%	10%	0.048/% RH, 290%(ΔR/R)	20 s/-	[30]
PET mesh	P(PEGMA-*co*-BPA) copolymer brush	Transmission	25–99%	1%	0.0423 mV/% RH, 96.8%(ΔV/V)	RH-dependent	This work

## Data Availability

Not applicable.
